# Alpha Hemolysin Induces an Increase of Erythrocytes Calcium: A FLIM 2-Photon Phasor Analysis Approach

**DOI:** 10.1371/journal.pone.0021127

**Published:** 2011-06-16

**Authors:** Susana Sanchez, Laura Bakás, Enrico Gratton, Vanesa Herlax

**Affiliations:** 1 Laboratory for Fluorescence Dynamics, University of California Irvine, Irvine, California, United States of America; 2 Microscopy Unit, Fundación CNIC-Carlos III, Centro Nacional de Investigaciones Cardiovasculares, Madrid, España; 3 Instituto de Investigaciones Bioquímicas La Plata (INIBIOLP), CCT- La Plata, CONICET, Facultad de Ciencias Médicas, Universidad Nacional de La Plata, La Plata, Buenos Aires, Argentina; 4 Departamento de Ciencias Biológicas, Facultad de Ciencias Exactas, Universidad Nacional de La Plata, La Plata, Buenos Aires, Argentina; Cornell University, United States of America

## Abstract

α-hemolysin (HlyA) from *Escherichia coli* is considered as the prototype of a family of toxins called RTX (repeat in toxin), a group of proteins that share genetic and structural features. HlyA is an important virulence factor in *E. coli* extraintestinal infections, such as meningitis, septicemia and urinary infections. High concentrations of the toxin cause the lysis of several cells such as erythrocytes, granulocytes, monocytes, endothelial and renal epithelial cells of different species. At low concentrations it induces the production of cytokines and apoptosis. Since many of the subcytolytic effects in other cells have been reported to be triggered by the increase of intracellular calcium, we followed the calcium concentration inside the erythrocytes while incubating with sublytic concentrations of HlyA. Calcium concentration was monitored using the calcium indicator Green 1, 2-photon excitation, and fluorescence lifetime imaging microscopy (FLIM). Data were analyzed using the phasor representation. In this report, we present evidence that, at sublytic concentrations, HlyA induces an increase of calcium concentration in rabbit erythrocytes in the first 10 s. Results are discussed in relation to the difficulties of measuring calcium concentrations in erythrocytes where hemoglobin is present, the contribution of the background and the heterogeneity of the response observed in individual cells.

## Introduction

Alpha hemolysin from *Escherichia coli* (HlyA) is generally considered the prototype of a large family of pore-forming toxins, named RTX, which are produced by gram-negative pathogens [1], [2]. In addition to their direct cytotoxic capability, pore forming toxins can trigger cellular responses that may produce important long term effects in the mammalian host organism. Many of these responses are triggered by the uncontrolled flux of monovalent and divalent ions across the plasma membrane [3], [4].

HlyA secreted from uropathogenic *E. coli* exerts a dual action on renal proximal tubule cells; sublytical concentrations induce host defense responses such as secretion of interleukins, while high concentrations cause irreversible cell damage [5]. In this system the deregulation of Ca^2+^ channels by HlyA is postulated to produce intracellular periodic low frequency calcium oscillations which further activate the pro-inflammatory cytokines IL-6, IL-8 [6].

However, Koschinski et al. reported that the Ca^+2^ oscillations induced by subcytolytic concentrations of HlyA, were not periodic, as expected from channel mediated oscillation, and did not respond to the Ca^2+^ channel blocker nifedipine. Furthermore, patch clamp experiments revealed temporal congruence between pore formation and Ca^2+^ influx. All together, this group concluded that the open/close status of HlyA pores is the trigger of nonperiodic Ca^2+^ oscillations in mammalian cell and not the deregulation of Ca^2+^ channels [7].

Circulating erythrocytes are among the most abundant cells contributing to almost 10% of cell volume in an adult human organism. They are easily accessible and could be functionally analyzed in any detail *ex vivo*. On the other hand, erythrocytes were the first cells described as susceptible targets to HlyA. Many papers have been published about the hemolytic mechanism of the toxin [7], [8], [9], [10], [11], [12], but only a few of these refers to the sublytical effects that may take place before hemolysis occurs [13], [14].

The aim of this paper is to study changes in the erythrocytes calcium concentration at sublytical concentration of the toxin. Although, erythrocytes seem to be an ideal system to measure free calcium due to the absence of organelles, they are rather problematic due to their auto-fluorescence, the presence of hemoglobin and the absorption properties of the intracellular milieu. Internal calibration and radiometric measurements are a common strategy used to overcome some of these experimental constraints [15]. A different approach is the use of fluorescence lifetime measurements. In this approach, the fluorescent dye shows different lifetime values depending on whether it is free or bound to Ca^+2^ and the equilibrium between these two species will be determined by the calcium concentration [16]. Since the fluorescence lifetime is an intrinsic property to the fluorescent molecule [17], lifetime measurements are independent of the dye concentration and the uneven labeling of the cells, a parameter which must be considered in fluorescence intensity measurements. The analysis of the FLIM data can be rather complex especially in cells and on an image [18]. We used the phasor plot analysis method, which was previously shown to simplify the analysis in both FRET [19] and ion determination [16], [20] experiments *in vivo*.

In this report we used Fluorescence Lifetime Imaging Microscopy (FLIM) and the phasor plot analysis to calculate the Ca^2+^ concentrations inside the erythrocytes and the changes produced after the addition of HlyA. Our results indicate that there is an average increase in internal Ca^+2^ concentration in the erythrocytes after interaction with HlyA and before hemolysis occurs. The analysis of the calcium concentration of single erythrocytes shows that the increase observed after addition of HlyA depends on the initial Ca^+2^ concentrations, which is different for each erythrocyte.

## Material and Methods

### Blood and chemicals

Rabbit blood was obtained from Colorado Serum Company (Colorado, USA). Erythrocytes were washed by centrifugation (1500 g, 10 min) with TC buffer (tris 20 mM, 150 mM NaCl, pH = 7.4). This procedure was repeated several times until the supernatant remained clear.

The calcium indicator, Calcium green-1 AM was obtained from Molecular Probes (USA) and ionomycin from Sigma-Aldrich (USA).

### Protein Purification

Cultures of *E. coli* WAM 1824 [9] were grown to late log phase in Luria-Bertani medium to an absorbance at 600 nm of 0.8–1.0. Cells were pelleted, and the supernatant was concentrated and partially purified by precipitation with 20% cold ethanol. The precipitate containing the protein was collected by centrifugation (1 h, 14,500 g in a Sorvall centrifuge, rotor SSA 34) and then resuspended in TC buffer. SDS-PAGE analysis of this preparation showed a main band at 110 kDa corresponding to more than 90% of the total protein. Proteins of lower molecular mass were removed by dialysis (membrane cutoff, 30 kDa). The protein was stored at −70°C in 20 mM Tris, pH 7.4, 150 mM NaCl, and 6 M guanidine hydrochloride (TCGn).

### Hemolytic Assays

For the hemolytic assays, an aliquot of toxin was serially diluted in TC buffer containing 10 mM CaCl_2_ on a 96-well microtiter plate. One hundred microliters of the diluted suspensions was mixed with 100 microliters of standardized erythrocytes, and the mixture was incubated at 37°C for 30 min. The absorbance of supernatants was read at 412 nm [21]. The standardization of the rabbit erythrocytes (RRBC) was done just before the assay. The erythrocytes were washed in 0.9% NaCl and then diluted to 12.5 µl in 1 ml of distilled water to give an absorbance reading of 0.6 at 412 nm [22].

### Measurement of the intracellular free Ca^2+^ concentration

#### Cell preparation

Intracellular free Ca^2+^ levels of single cells were monitored using the Ca^+2^ sensitive fluorescent dye Calcium Green™-1 AM (CaG-1). Standardized rabbit erythrocytes were incubated with 5 µM CaG-1 in the presence of 16 µM of Pluronic Acid at 37°C for 30 min in the dark. The non-ionic detergent was used to assist in dispersion the non-polar AM ester in aqueous media. Cells were then washed in TC buffer to remove any dye, and resuspended in the corresponding volume of TC buffer to maintain standardization of the erythrocytes.

For microscopy measurements, cells were placed on poly-L-lysine coated cell culture dishes. Control images for erythrocytes were taken (t = 0) before the addition of HlyA. Next, the buffer was carefully removed from the dish and 0.82 nM of HlyA in TC buffer containing 10 mM CaCl_2_ was added. The kinetics of the interaction was followed taking images every 13 sec (average of 5 images 2.6 sec each) until hemolysis occurred. Controls for changes in Ca^2+^ during the acquisition time were done using labeled RRBC and adding only buffer. The control for auto fluorescence was achieved using unlabeled RRBC.

### Instrumentation

FLIM data were acquired in a two photon microscope equipped with a Becker & Hickl 830 card (Becker and Hickl, Berlin). The two-photon excitation scanning fluorescence microscope used in these experiments was assembled at the Laboratory for Fluorescence Dynamics [23]. Briefly, the laser source is a Ti:Sapphire laser (Mai Tai HP, Spectra Physics, Newport Beach, CA) producing 100 fs pulses at a repetition rate of 80 MHz. The laser light is coupled to a Zeiss M135 microscope equipped with galvano-scanners (Model 6350; Cambridge Technology, Cambridge, MA). We used a Zeiss 40×/1.2 NA) water immersion objective. A BG39 barrier filter was used to block the near-IR light. Fluorescence counts were detected with a hybrid HPM-100-40 detector from Becker & Hickl GmbH (Berlin, Germany). Data were acquired and processed by the SimFCS software developed at the Laboratory for Fluorescence Dynamics. For referencing the lifetime data and to account for the instrument response, a fluorescein solution at pH = 10 was used as a standard with a known lifetime of 4.05 ns. The excitation wavelength for CaG-1 was 960 nm and the power of the laser was set at 84 mW.

### Method

The phasor plot approach was used to analyze the FLIM data using the data analysis program SimFCS (Laboratory for Fluorescence Dynamics, Irvine, CA). The application of this methodology to the determination of calcium concentrations in skin [16] has been described in detail, therefore here only the basic concepts will be described.

The phasor is a frequency domain representation of the fluorescence lifetime at a single frequency. The phasor analysis approach is based on the transformation of the fluorescence decay histogram I(t) into its sine and cosine components and does not require fitting of the data to exponential decays.

**Figure 1:**
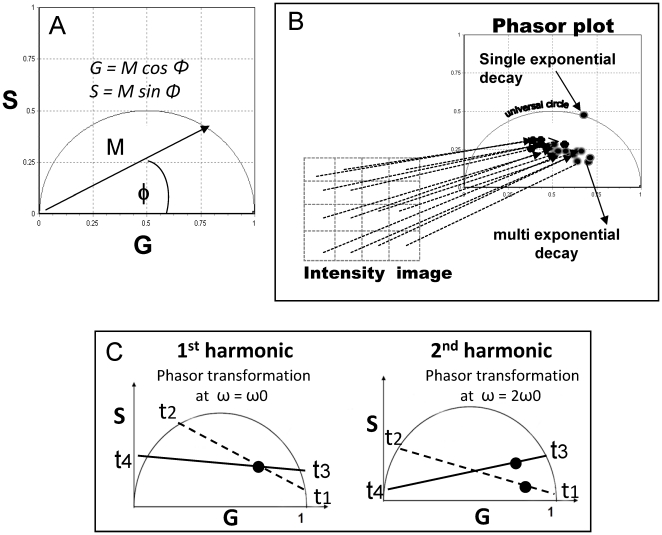
The phasor representation method. The phasor plot coordinates are S = M cos ϕ and G = M sin ϕ (Figure 1A), with ϕ being the phase delay between excitation and emission and M the demodulation (**A**). Each fluorescent species, independently of the number of exponentials needed to determine its decay, is represented by a point (phasor) with (S,G) coordinates. From an image, the lifetime will be determined at each pixel and the corresponding phasor will be represented on the phasor plot (**B**). The phasor analysis can be performed in any harmonic of the original modulation frequency. If two phasors are the linear combination of different pair of phasors, for example t_1_-t_2_ and t_3_-t_4_, they may overlap in the first harmonic (**C left**), but may be separated at higher harmonics (**C right**) **(see Methods)**.

Data are displayed in a polar plot (phasor plot) (Figure 1A) with coordinates S = M cos ϕ and G = M sin ϕ (Figure 1A), with ϕ being the phase delay between excitation and emission, and M the demodulation [24], [25], [26], [27], [28].

Phase delay and modulation can be related to the lifetime using the following equations:
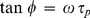
(1)

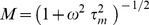
(2)Where ω is the angular modulation frequency (equal to 2π*f*, where *f* is the modulation frequency). The phasor analysis can be performed with any harmonics of the original modulation frequency. Without changing the lifetime value, the use of a different harmonics changes the location of the corresponding phasor in the plot because the values ϕ and M change (equations 1 and 2). This property can be very useful to separate phasors if they belong to different species. If two phasors are the linear combination of different pair of phasors, for example t_1_-t_2_ and t_3_-t_4_ (Figure 1C) they may overlap in the first harmonics as shown in Figure 1C left, but they can be separated at higher harmonics (Figure 1C right), since the position of each individual phasor changes with respect to the other.

In the phasor representation each fluorescent species, independently of the number of exponentials needed to determine its decay, is represented by a point (phasor) with (S,G) coordinates.

From an image, the lifetime will be determined at each pixel and the corresponding phasor will be represented on the phasor plot (Figure 1B). Thus, the phasor plot contains as many phasors as the number of pixels in the image, for example the phasor plot of 256×256 pixels will contain 577,536 phasors. Single exponential lifetime decays will be located on the universal circle of the phasor plot, and non mono-exponential decays will be located inside the universal circle (Figure 1B). The phasor analysis does not require accurate determination of the underlying lifetime components, the location and the relationship between the phasors will give information about the system [18].

### Data analysis

#### Calculation of Ca^2+^ concentration

A detailed derivation of the equations involved in the calcium concentration calculations using Calcium Green 5N have been previously published [16]. Similarly, for Calcium Green 1 (CaG-1), at equilibrium:
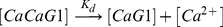
(3)


The calcium bound and calcium free species of CaG-1 have different lifetime decays and the phasors for each species can be called **p_b_** (phasor for the bound specie) and **p_f_** (phasor for the free species) respectively. Different concentrations of calcium will move the equilibrium as shown in eq. 3 and the fraction of bound and free dye will reflect this shift. In the phasor plot the phasors for solutions with different calcium concentrations will align between **p_b_** and **p_f_** according to the fraction of each component [16].

The fraction of the dye bound to Ca^2+^ in terms of phasors is defined as
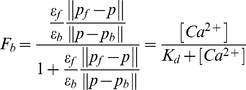
(4)Where **p** corresponds to the unknown phasor, **ε_f_/ε_b_** is the quantum yield ratio of the free and bound forms and **K_d_** is the dissociation constant for equilibrium 1.

The phasors for the free (**p_f_**) and bound (**p_b_**) CaG-1 species were determined using the samples with the lowest and the highest calcium concentrations, respectively. For the calibration solution these samples correspond to the standard commercial solutions and in erythrocytes they correspond to the samples containing low and high calcium concentration, with erythrocytes pretreated with Ionomicine. The lifetime data were analyzed using the program SimFCS (Laboratory for Fluorescence Dynamics, University of California, Irvine). Once **p_f_** and **p_b_** for a given sample were identified and entered into the SimFCS routine, the program calculates the fraction of the dye bound to calcium (Fb).

## Results

### Determination of K_d_ for Calcium Green™-1

Calcium calibration solutions (Molecular Probes) of 0, 17, 38, 65, 100, 150, 225, 352 and 602 nM were used to obtain the K_d_ of Calcium Green™-1 (CaG-1), hexapotassium salt (cell impermeant). Lifetime measurements for the individual solutions were done using the same microscope set up used for cell measurements. The excitation wavelength was 960 nm and the measurements were performed in duplicate. The phasors for the bound (p_b_) and free species (p_f_) were determined using the lowest (0 nM) and the highest (602 nM) calcium concentration standard solution. The intermediate calcium concentrations lined up between these two extremes as reported before for Calcium Green 5N [16]. The K_d_ was determined at 50% bound dye and it was corrected for differences in the quantum yield between the free and bound species. We determined a K_d_ value of 76 nM for CaG-1. The K_d_ inside the cells must be approximate using the K_d_ found in buffered solutions [16].

### Determination of Calcium inside the RRBCs

#### Dealing with auto-fluorescence (phasor analysis in the second harmonics)

The main challenge when measuring cytosolic Ca^2+^ in erythrocytes is to avoid the signal produced by hemoglobin [15]. We used CaG-1 and two-photon excitation at 960 nm. At this wavelength the excitation of hemoglobin was minimal.

**Figure 2:**
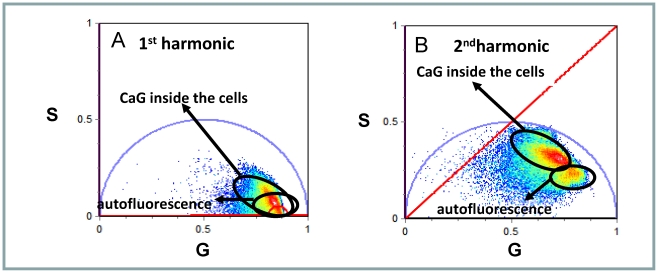
Separation of auto fluorescence from CaG-1 signal in RRBC. Phasor representation of FLIM images acquired on RRBC incubated with and without CaG-1. For comparison purposes, the same power and the same integration time were used to obtain the images from both samples. The auto fluorescence corresponds to the signal obtained from the RRBC without CaG-1, Data were analyzed calculating the phasors at the basic laser frequency (80 MHz) (**A**) and at the second harmonic of it (160 MHz) (**B**). Using the second harmonic the cluster of phasors from the auto fluorescence can be separated from the CaG-1 phasors and the concentration of Ca^+2^ inside the RRBC can be determined.

In RRBCs we found that the cluster of phasors corresponding to the auto-fluorescence (erythrocytes without calcium green) and to the desired signal (CaG-1 inside the erythrocytes) are located inside the phasor universal circle (Figure 2A), indicating that both lifetimes are non single – exponential. These two groups of phasors overlapped (Figure 2A). Overlap of the auto- fluorescence signal and the signal of interest is a common and very difficult problem to solve when working with fluorescence intensity or traditional lifetime analysis; however the phasor analysis offers the possibility to separate the non single exponential phasors based on their mono exponential component. If the phasors overlapping in the 1^st^ harmonic originate from the linear combination of different pairs of single exponential phasors, analysis at a different frequency will separate them and allow the biological analysis to be performed (methods). Figure 2B shows the analysis of the same data shown in Figure 2A using a frequency of 160 MHz (second harmonic of the original frequency of acquisition). At this frequency the cluster of phasors from the dye inside the cells can be easily separated from the phasors corresponding to the auto-fluorescence, allowing the analysis of the CaG-1 signal without interference from the auto fluorescence. Hence all the analyses were performed at this frequency.

### Calcium calibration inside the RRBC

For the concentration measurement of any ion, the main experimental challenge is the calibration of the dye response to the ion concentration inside the cells. For the methodology used here, the phasor for the free (**p_f_**) and bound (**p_b_**) species inside the erythrocytes have to be determined. **p_f_** and **p_b_** measured inside the RRBC may be different from the values measured using calcium calibration kits solutions (buffers with precise amounts of calcium). This is a consequence of the fact that the conditions inside cells (ionic strength, pH, viscosity, etc.) are different from those in buffers [16], [29].

**Figure 3:**
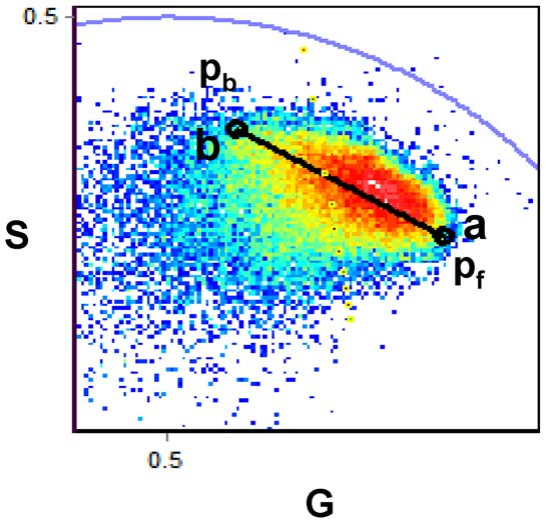
Calibration of CaG-1 inside the RRBC. Phasor plot representation of FLIM data acquired from sequential images of RRBC containing CaG-1, after the addition of 0.82 nM of HlyA and before hemolysis occurrence. Black dots represent the phasor of the bound (p_b_) and free (p_f_) species of CaG-1 inside the cells. The line is the internal calibration curve determined by using the phasors at the extremities and the K_d_ determined for the dye (79 nM).

The phasor approach allows us to calibrate the fluorescence response of CaG-1 to calcium inside the RRBC, provided that the calcium concentrations at the highest and lowest ends of the dye sensitivity range exist in the sample. Figure 3 shows the phasor plot from a set of six images corresponding to a time series after HlyA addition to RRBC. Phasors corresponding to the calcium-free (**p_f_**) and calcium-bound (**p_b_**) species can be identified as the extremes of the distribution and they are represented as black dots labeled as **a** and **b**, respectively, in Figure 3. These extremity phasors and the K_d_ previously determined, are used in SimFCS to calculate the calibration curve showed as a black line in Figure 3. To further validate our calibration, we determined the phasors of RRBC incubated with solutions containing 1 µM Calcium and 1 mM EGTA in the presence of Ionomicin (0.3 µM) to equilibrate the cell interior. The lifetime distribution measured for these samples lie at the lowest and highest extremes of the calibration curve. These two points define the limits for the calcium determination in the RRBC.

**Figure 4:**
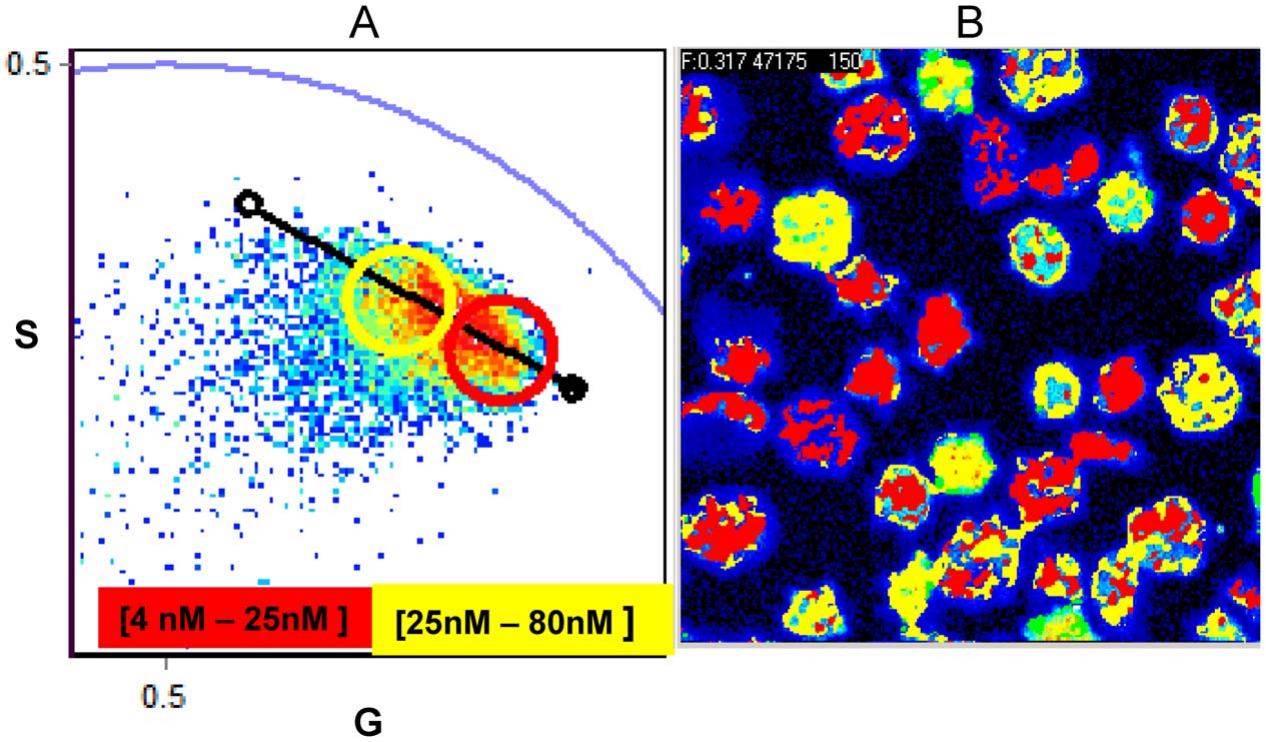
Calcium concentration on RRBC without stimulus. Phasor representation of FLIM data (**A**) and intensity image (**B**) of RRBC labeled with CaG-1 at 37°C. In the phasor plot two areas of calcium concentration have been defined and color coded as red for 4–25 nM and yellow 25–80 nM. The pixels corresponding to the phasors inside each colored circle in the phasor plot (**A**) are highlight in the intensity image (**B**) with the corresponding color. The black line and the dots at the extremities correspond to the internal calibration curve described in Figure 3 and in the text.

Figure 4 shows the intensity image of untreated RRBCs labeled with CaG-1 at 37°C with its corresponding phasor plot. Each pixel in the intensity image has its corresponding phasor in the phasor plot, and this correspondence is indicated by a color code. The black line is the calibration curve previously obtained. The image shown in Figure 4 clearly shows that in basal conditions the concentration of Ca^2+^ in the different cells is very heterogeneous, ranging from 4 to 80 nM. This concentration range was divided into two regions, one going from 4 to 25 nM and the other from 25 to 80 nM, using red and yellow to color the pixels in the image, respectively.

**Figure 5:**
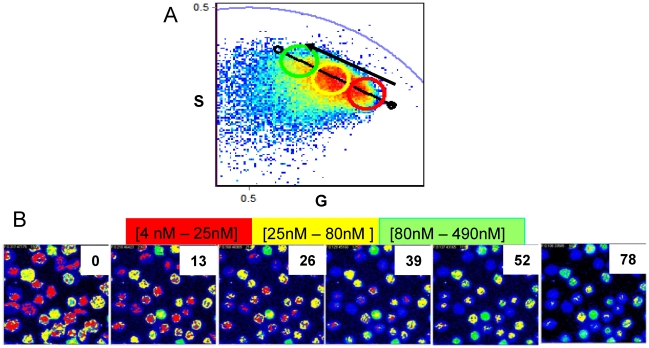
Calcium concentration changes after HlyA addition. Phasor representation of FLIM data (**A**) and the corresponding intensity images (**B**) of RRBC labeled with CaG-1 at 37°C after the addition of 0.82 nM of HlyA. Red and yellow concentration ranges are the same used in Figure 4, green concentration range goes from 80 to 490 nM. The black line and dots at the extremes represent the internal calibration curve determined for RRBC. The black arrow indicates the direction of movement experienced by the center of the phasor cluster after the addition of HlyA Numbers on the upper right of each image correspond to the time in seconds after HlyA addition.

Starting from this heterogeneous population HlyA was added to the RRBCs and FLIM images were taken at different times. Figure 5A shows the phasors corresponding to a series of 6 images taken every 13 seconds after the addition of 0.82 nM of HlyA. This toxin concentration (sublytical) corresponds to the one producing 40–50% hemolysis in 30 minutes and was determined using a hemolytic activity assay (methods).

**Figure 6:**
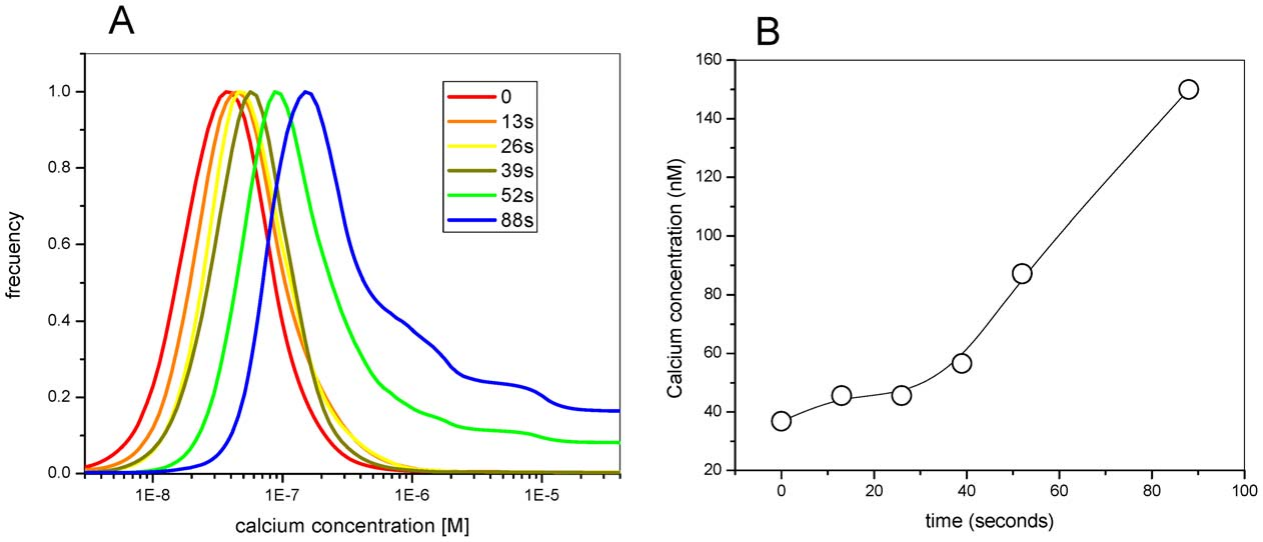
Increment of internal calcium after HlyA addition. Histograms of the phasor distributions that are shown in Figure 5A, for RRBC (**A**). The maximum of the histograms corresponds to the average of calcium concentration, which is plotted as function of time in RRBC (**C**).

Image analysis was performed until hemolysis occurred, as evidenced by the deformation of the RRBC and the release of all of the internal content to the media. The color code in Figure 5A is the same used in Figure 4 and is related to the calcium concentration. By the time of interaction with HlyA increases the cluster of phasor corresponding to the image moved in the direction of increasing calcium concentration as indicated by the black arrow (Figure 5A). We have added a new color circle (green) corresponding to a calcium concentration range from 80 to 490 nM. Figure 6A shows the histograms of the cluster of phasors associated with the time series of images shown in Figure 5B, and Figure 6B shows the changes of calcium concentration with time. An average increase in calcium concentration is observed after the addition of HlyA: (i) for the first 40 s the average calcium concentration increased from 36 to 57 nM, (ii) for the following 40 seconds another increase which takes the calcium concentration to 150 nM. The kinetics of the two processes showed slopes of 0.33 and 1.86 nM/s.

**Figure 7:**
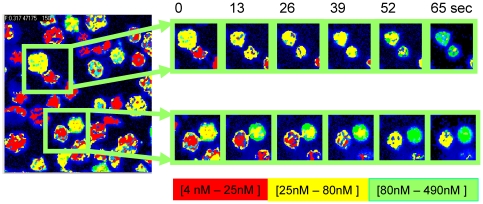
Single cell increase in calcium.

Using the SimFCS program we were able to follow the changes in the calcium concentration in each individual cell as shown in Figure 7. From the image it can be appreciated that most of the RRBC present an increase in calcium concentration. The extent of the increase depends on the initial calcium concentration of each particular cell.

## Discussion

In this manuscript, there are two important topics that have to be discussed: (i) the quantification of calcium increment by FLIM and (ii) the increment of intracellular calcium concentration in RRBC due to its interaction with sublytical concentration of HlyA.

Calcium homeostasis plays an important role in the physiology and pathophysiology of erythrocytes [30], and several methodologies have been developed to measure this ion. Fluorescence methodologies have been used to qualitatively measure calcium in red blood cells [30], [31], however, to the best of our knowledge there are no reports of methods for quantification of intra-erythrocyte calcium concentrations. This quantification is challenging due to the auto fluorescence that erythrocytes show when they are irradiated with UV or visible light, normally used to excite the fluorescent dyes employed for calcium determinations [32]. This auto- fluorescence might be due to the high NADH and NADPH content or to the formation of bilirubin isomers as consequence of the UV irradiation [33], therefore it varies in the different erythrocyte populations depending on their metabolic state.

Two-photon excitation using 960 nm, minimizes the auto fluorescence and the excitation of hemoglobin is minimal at this wavelength. However, even under this optimal condition, the phasor cluster from the auto fluorescence overlaps with the phasors corresponding to the CaG-1 inside the erythrocytes when the two samples are measured under the same conditions of power and integration time. To eliminate this problem we performed the phasor analysis using a frequency of 160 MHz (second harmonics of the original frequency acquisition). Working at this frequency the phasors could be separated allowing us to measure the intraerythrocyte calcium concentration without the interference of the background.

An important increment of calcium concentration in RRBC was observed after their interaction with HlyA. This calcium increment was very fast, starting after the first 10 sec of incubation with the toxin, much earlier than the beginning of hemolysis which occurs after 80 s. The increment of calcium in RRBC was biphasic for the first 40 seconds showing an initial increase in the average calcium concentration from 36 to 57 nM, and for the following 40 seconds another, faster increase that raised the calcium concentration to 150 nM. The influx rates were 0.33 and 1.86 nM/s, respectively, suggesting a different mechanism for calcium increase. One can think of a scenario where in the first increment of calcium is produced by activation of non- selective cation channels activated by the toxin. This initial rise in calcium concentration might induce membrane reorganization by activation of several enzymes (i.e., flippase) that facilitate the pore formation allowing a second abrupt increment of calcium, prior to hemolysis. This hypothesis needs further investigation.

Something to highlight is the fact that with FLIM analysis, the increment of calcium can be followed in time, at each individual cell of the image. A simple observation of Figure 5 shows that, before the interaction of the RRBC with the toxin, the calcium content in the individual erythrocytes is heterogeneous. After the interaction most of the cells showed an increase in calcium regardless of the basal calcium concentration, however some cells did not respond.

Skals *et al.* demonstrated that shrinkage and crenation of erythrocytes occur after the internal calcium concentration increased two fold within 70 s [13]. In this work we confirmed that the calcium increment is very fast (80 s) and we quantified this increment which occurs in two phases with an average total change in calcium concentration from 36 to 150 nM. Several processes can be connected to the increase of intracellular calcium concentration in erythrocytes due to its interaction with the toxin. For example phosphatidylserine (PS) externalization may be induced by the toxin [13]. Phospholipid asymmetry is maintained by an ATP- dependent aminophospholipid translocase (flippase), which returns externalized PS to the inner leaflet [34], [35]. On the other hand, asymmetry is lost by activation of the phospholipid scramblase, which causes non-specific bidirectional transport of phospholipids across RBC membrane [36], [37]. It has been postulated that both inhibition of flippase and activation of scamblase are required for PS externalization in RBC [38]. However, the calcium concentrations involved in the inhibition of flippase and the activation of scamblase are different. Flippase is inhibited at relatively low, but nevertheless elevated calcium concentration (90 nM) [39], while scramblase is activated at much higher calcium concentration (10–50 µM) [40]. Considering our results, only flippase is inhibited, so the externalization of PS may also be a consequence of the formation of the proteolipidic pre-pore, where membrane phospholipd reorganization occurs [12], [41], but this hypothesis requires further investigation.

In summary, two main contributions are presented in this work: (i) A methodology to measure the calcium concentration inside erythrocytes. This methodology involves: a lifetime sensitive calcium fluorescent dye (calcium green-1), fluorescence lifetime imaging microscopy (FLIM), two-photon excitation and the phasor analysis for lifetime decay. (ii) From the biological point of view we have demonstrated that as a result of the interaction of HlyA with rabbit erythrocytes, the intracellular calcium increases almost four fold in a biphasic manner, within 80 sec, and before hemolysis occurs. This finding raises several questions (beyond the scope of this article) on the mechanism(s) involved in the calcium concentration increment. The quantification of the intracellular calcium concentration is necessary for a more exhaustive analysis of transduction pathways in different cells. Related to HlyA, the next step is to study if the influx of calcium to erythrocytes is directly through the pore formed by the toxin or if the toxin activates a preexistence channel.

These findings should encourage future research about calcium signaling in erythrocytes, and allow for an exhaustive study of the mechanism of action of the toxin, leading to clarification of the infection process produced by *E. coli*.
